# Alterations in early auditory evoked potentials in multiple sclerosis patients

**DOI:** 10.1016/S1808-8694(15)30775-8

**Published:** 2015-10-19

**Authors:** Tania Mara Assis Lima, Aline Nascimento Crato, Patrícia Cotta Mancini, Lília Correa Simões, Denise Utsch Gonçalves

**Affiliations:** 1 PhD in Otorhinolaryngology, Adjunct Professor in the Department of Otorhinolaryngology at FM UFMG; 2 Speech and Hearing Therapist; 3 MSc in Linguistics, Associate Professor in the Department of Speech and Hearing Therapy at FM UFMG; 4 Speech and Hearing Therapist in the Audiology Ward at Hospital das Clínicas da UFMG; 5 PhD in Tropical Medicine, Adjunct Professor in the Department of Otorhinolaryngology at FM UFMG. UFMG Medical School, Hospital das Clínicas da UFMG

**Keywords:** multiple sclerosis, auditory evoked potentials, brainstem

## Abstract

Alterations in early auditory evoked potentials (EAEP) in individuals with demyelinating disease are suggestive of lesions in the brainstem.

**Aim:**

this study aims to evaluate the prevalence of hearing disorders and altered EAEP in multiple sclerosis (MS) patients.

**Materials and method:**

sixteen female and nine male patients with a defined diagnosis of multiple sclerosis took part in this study. All individuals underwent hearing and EAEP tests. The wave forms were categorized according to Jerger (1986).

**Results:**

fifty EAEP tests were carried out; 70% were classified as type I (normal response) according to Jerger's criteria. Altered EAEP results in at least one ear were classified into types II, III, IV or V according to Jerger. Females accounted for 31.25% of alterations, and males 44.44%, adding up to 36% of all cases.

**Conclusions:**

these findings stress the importance of looking at EAEP in cases where there is suspicion of demyelinating disease and in patients with a defined diagnosis for MS.

## INTRODUCTION

Brainstem auditory evoked potentials (BAEP) are short latency potentials generated in the auditory nerve and in the auditory pathways of the brainstem as the cochlea is activated in frequencies ranging between 2000 and 4000Hz after the introduction of sound stimuli. They are characterized by a series of positive peak waves that come up within the first 10 milliseconds (ms) and that represent the summation of the neural activity of one or more sites. The more important waves are the rising ones, namely I, III, and V^1^.

BAEP results are altered in neurologic disorders introduced by tumors, diffuse lesions, demyelinating diseases, or functional disorders^2^.

Multiple sclerosis (MS) is the most common type of demyelinating disease and the main cause of neurologic damage in young adults. This is a slow, progressive disease that features periods of symptom exacerbation and remission^3^ and huge variation between individuals, depending on the site of the lesion^4^.

BAEP tests in individuals with demyelinating disease are important to assess central nervous system involvement^5^. The incidence of altered results in MS patients reported in the literature varies considerably. Wave I is almost always normal in patients without hearing loss. The anomalies found in patients with MS include: prolonged wave latency and intervals between peaks; reduced amplitude on wave V; reduced wave V/I amplitude ratio; absent wave V^6^.

The goal of this study is to assess the incidence of hearing disorders and altered BAEP test results in individuals with multiple sclerosis, looking at hearing thresholds and absolute latencies, intervals between peaks, wave characteristics, morphology and reproducibility.

## MATERIALS AND METHOD

This study was approved by the UFMG-COEP Ethics Committee under permit 052/2004. Sixteen female and nine male patients from the Multiple Sclerosis Research Center (Centro de Investigação em Esclerose Múltipla–CIEM) were enrolled in the study. All had clinically defined diagnosis for multiple sclerosis according to the criteria set by Poser^7^. Clinical manifestations were categorized according to the principles proposed by Lubin and Reingold ^8^. The female patient group's age ranged between 33 and 53 years (mean 42.6 years) while the male patient group was aged between 24 and 56 years (mean 38 years).

All patients were interviewed and underwent clinical ENT examination, tympanometry, tone audiometry, and BAEP tests.

The criteria defined by Russo^9^ were used to sort tympanometry results. Audiometry results were classified according to the principles defined by Davis and Silverman^10^.

On the BAEP tests we used a sound stimulus of 1024 clicks introduced by a TDH-39 rarefied polarity supra-auricular transducer in 100ms pulses, at 60dB of sound pressure level above the psychoacoustic threshold of each subject. Stimuli were presented in one ear at a time, masked by white noise in the contralateral ear at 30dB SPL.

High pass filters at 3000Hz and low pass filters at 100Hz were used. Clicks were introduced at a rate of 13.1/sec on the right ear and then on the left ear.

The criteria defined by Munhoz^11^ were used to define normal BAEP findings.

Waves were categorized as defined by Jerger^12^:

Type I: normal response; all waves (I-V) had normal latencies and between peak intervals.

Type II: prolonged interval I-V.

Type III: degraded wave form; poor morphology; peaks hard to tell apart and/or reduced amplitude; and/or altered amplitude ratios.

Type IV: absent last waves; absent last waves, first waves present with normal latency.

Type V: extremely altered response or only wave I normal.

## RESULTS

Fifty-six percent of the 25 patients enrolled in the study had acute remissive MS, as shown in Table 1. Sixty-eight percent of the patients had tone hearing thresholds within normal range, and other cases of hearing loss did not interfere with BAEP acquisition (Table 2, Table 3).

**Table 1:** MS Clinical forms according to the Lublin and Reingold Classification

					Gender				
		Female			Masculino			Total	
Clinical form			Male	N		%	N		%
Remission flare			62,50	4		44,44	14		56
Progressive flare	1		6,25	2		22,22	3		12
Secondary progressive	0		0	0		0	0		0
Primary progressive	5		31,25	3		33,33	8		32
TOTAL	16		100	9		100	25		100

N = number

**Table 2:** Hearing loss classification as to intensity according to Davis and Silverman

				Females							Males			
		RE				LE			RE				LE	
Classification	N		%		N		%	N		%		N	%	
Normal	12		75		11		68,75	6		66,66		6	66,66	
Mild	1		8,33		1		6,25	2		22,22		2	22,22	
Moderate	3		16,66		4		25	1		11,11		1	11,11	
Severe	0		0		0		0	0		0		0	0	
Profound	0		0		0		0	0		0		0	0	
TOTAL	16		100		16		100	9		100		9	100	

N = number RE: Right ear LE: Left ear

**Table 3:** Tonal audiometry results according to Davis e Silverman

		Females			Males			Total	
Audiometry	N		%	N		%	N		%
Normal	11		68,75	6		66,66	17		68
Altered	5		31,25	3		33,33	8		32
TOTAL	16		100	9		100	25		100

N = number

From the 50 BAEP tests done, 70% were categorized as type I. Prolonged intervals between peaks I-V were found in 6.25% of the female subjects and 11.11% of the male patients, and they were categorized as type II. Wave form degradation and poor morphology was found in 5.55% of the male group, and they were categorized as type III. Type IV was not found. Type V, with more altered tracings, occurred in 18.75% of females and 22.22% of males (Table 4).

**Table 4:** Classification of wave types according to Jerger

		Females			Males			Total	
Type I	N 24		% 75,00	N 11		% 61,11	N 35		% 70
Type II	2		6,25	2		11,11	4		8
Type III	0		0	1		5,55	1		2
Type IV	0		0	0		0	0		0
Type V	6		18,75	4		22,22	10		20
TOTAL	32		100	18		100	50		100

N = number

Subjects with types II, III, and IV were considered to have altered BAEP results in at least one ear. Thirty-six percent of the patients had altered BAEP results, with incidences of 31.25% among females and 44.44% among males (Table 5).

**Table 5:** Classification of BAEP in both genders

		Females			Males			Total	
	N		%	N		%	N		%
Normal	11		68,75	5		55,55	16		64
Altered	5		31,25	4		44,44	9		36
TOTAL	16		100	9		100	25		100

N = number

Individual values of absolute latency and between peak intervals in MS patients can be seen on Chart 1, Chart 2.

**Chart 1:** Individual values of absolute latencies and interpeak intervals of female individuals

		Waves						Interpeak intervals			
I		III		V		I-III		III-V		I-V	
RE	LE	RE	LE	RE	LE	RE	LE	RE	LE	RE	LE
1,52	1,56	3,44	3,40	5,52	5,72	1,92	1,84	2,08	2,32	4,00	4,16
1,48	1,76	3,28	3,52	5,20	5,72	1,80	1,76	1,92	2,20	3,72	3,96
1,60	1,60	3,48	3,52	5,48	5,60	1,88	1,92	2,00	2,08	3,88	4,00
1,68	1,72	3,88	3,96	6,00	5,92	2,20	2,24	2,12	1,96	4,32	4,20
1,56	1,56	3,88	3,80	5,28	4,88	2,32	2,24	1,40	1,08	3,72	3,32
1,44	1,76	3,44	3,44	5,16	5,72	2,00	1,68	1,72	2,28	3,72	3,96
1,56	1,72	3,68	3,52	5,56	5,72	2,12	1,80	1,88	2,20	4,00	4,00
1,64	1,60	3,88	3,80	5,84	5,92	2,24	2,20	1,96	2,12	4,20	4,32
1,68	1,56	3,84	3,80	5,36	5,36	2,16	2,24	1,52	1,56	3,68	3,80
–	–	–	–	–	–	–	–	–	–	–	–
–	–	–	–	–	–	–	–	–	–	–	–
1,64	1,80	3,48	3,60	6,24	5,88	1,84	1,80	2,76	2,28	4,60	4,08
1,60	1,40	4,16	3,60	6,24	5,28	2,56	2,20	2,08	1,68	4,64	3,88
1,12	1,16	2,80	2,84	4,64	4,76	1,68	1,68	1,84	1,92	3,52	3,60
1,36	1,36	3,56	3,48	5,60	5,44	2,20	2,12	2,04	1,96	4,24	4,08

RE: Right Ear LE: Left Ear

**Chart 2:** Individual values of absolute latencies and interpeak intervals of males

		Waves						Interpeak Intervals			
I		III		V		I-III		III-V		I-V	
RE	LE	RE	LE	RE	LE	RE	LE	RE	LE	RE	LE
1,52	1,56	3,76	3,64	5,48	5,48	2,24	2,08	1,72	1,84	3,96	3,92
1,60	1,44	3,64	3,60	5,56	5,60	2,04	2,16	1,92	2,00	3,96	4,16
–	–	–	–	–	–	–	–	–	–	–	–
1,12	1,12	3,28	3,20	5,32	5,32	2,16	2,08	2,04	2,12	4,20	4,20
1,48	1,60	3,80	4,52	5,96	6,80	2,32	2,92	2,16	2,28	4,48	5,20
1,48	1,64	3,84	3,72	5,80	5,80	2,36	2,08	1,96	2,08	4,32	4,16
1,40	–	–	–	–	–	–	–	–	–	–	–
1,40	1,24	3,76	3,84	5,12	5,92	2,36	2,60	1,36	2,08	3,72	4,68
1,48	1,48	3,64	3,84	5,80	5,92	2,16	2,36	2,16	2,08	4,32	4,44

RE: Right Ear LE: Left Ear

## DISCUSSION

Hearing loss in MS is sensorineural and bilateral in 85% of the cases^3^. In our study, tone audiometry results were altered in 32% (8) of the patients, with 87.5% having bilateral sensorineural hearing loss and 12.5% being affected by moderate left conductive hearing loss due to ear drum perforation. Jerger^12^ reported altered audiometry results in 39% of multiple sclerosis patients, all of whom affected by sensorineural hearing loss.

MS neurologic symptoms are many, and may vary from case to case and throughout the progress of the disease. In Brazil, Lana-Peixoto and Lana-Peixoto13 found brainstem alterations in 34% of the individuals with manifestations compatible with early MS and 45% with progressed disease. Papais-Alvarenga et al.^14^ found brainstem alterations in 28.4% of early MS patients and in 28.5% of patients with progressed MS. Tilbery et al.^15^ found signs and symptoms of brainstem alteration in 32% of patients with early MS and in 52% of patients with progressed disease.

Our study showed altered BAEP in 9 patients (36%) in one or both ears, 31.25% of whom were females and 44.44% males. In 66.7% of these patients, altered results were found in both ears. Jerger^12^ found 52% of BAEP alteration in one or both ears, 68.75% of whom had bilateral hearing loss. Santos et al.^16^ found altered unilateral and bilateral results in 58.62% of the patients, 60% in females and 56% in males.

The most frequently found alteration in our study was type V^13^, as it occurred in 18.75% of females and 22.22% of males. Jerger12 reported type II alteration in 6.5%, type III in 8%, type IV in 16.1%, and type V in 13% of patients. Santos^16^ reported type II in 32.8%, type IV in 5%, and type V in 7% of patients.

## CONCLUSION

Thirty-six percent of the 25 patients with clinically diagnosed MS had brainstem alterations as found in BAEP tests, thus stressing the importance of this test in examining patients clinically suspected for demyelinating disease.
